# Turning up the heat on essential *E. coli* genes

**DOI:** 10.15252/msb.202311933

**Published:** 2023-09-18

**Authors:** Arun Kumar, Peter C Stirling

**Affiliations:** ^1^ Terry Fox Laboratory BC Cancer Research Institute Vancouver BC Canada

**Keywords:** Biotechnology & Synthetic Biology, Metabolism, Microbiology, Virology & Host Pathogen Interaction

## Abstract

Temperature‐sensitive (TS) alleles create tunable thermoswitches to deplete essential cellular activities and are used to dissect gene function. In their recent study, Link and colleagues (Schramm *et al* 2023) use a CRISPR‐based approach to systematically create TS alleles across essential genes in *E. coli*.

## Graphical abstract

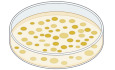

Other technologies for inducible disruption of essential gene function often rely on exogenous ligands or protein tagging (Yesbolatova *et al*, 2020). TS alleles on the contrary are simple to use and can model a range of activity levels by shifting the organism from permissive, to semipermissive, to restrictive temperatures. Working with a list of 346 *E. coli* genes that are essential in minimal glucose media, Schramm *et al* (2023) designed 50 amino acid mutations for each gene using the TSPred algorithm (Tan *et al*, 2014), focusing on mutations that would affect thermostability of the respective protein. With the designed guides, they used CRISPR knockin to create a strain library with > 15,000 edited *E. coli* strains. This collection contained > 6,000 hypomorphic alleles, which dropped out prior to the temperature shift. Of the remaining alleles, 1,269 behaved like TS alleles, and the authors isolated 94 for further characterization (Fig 1A). This approach was remarkably successful as in this first study the authors created new TS alleles for nearly one third of *E. coli* essential genes. Moreover, their pool contained > 1,200 putative alleles raising confidence that a systematic collection for all 346 genes could be produced using this approach.

**Figure 1:**
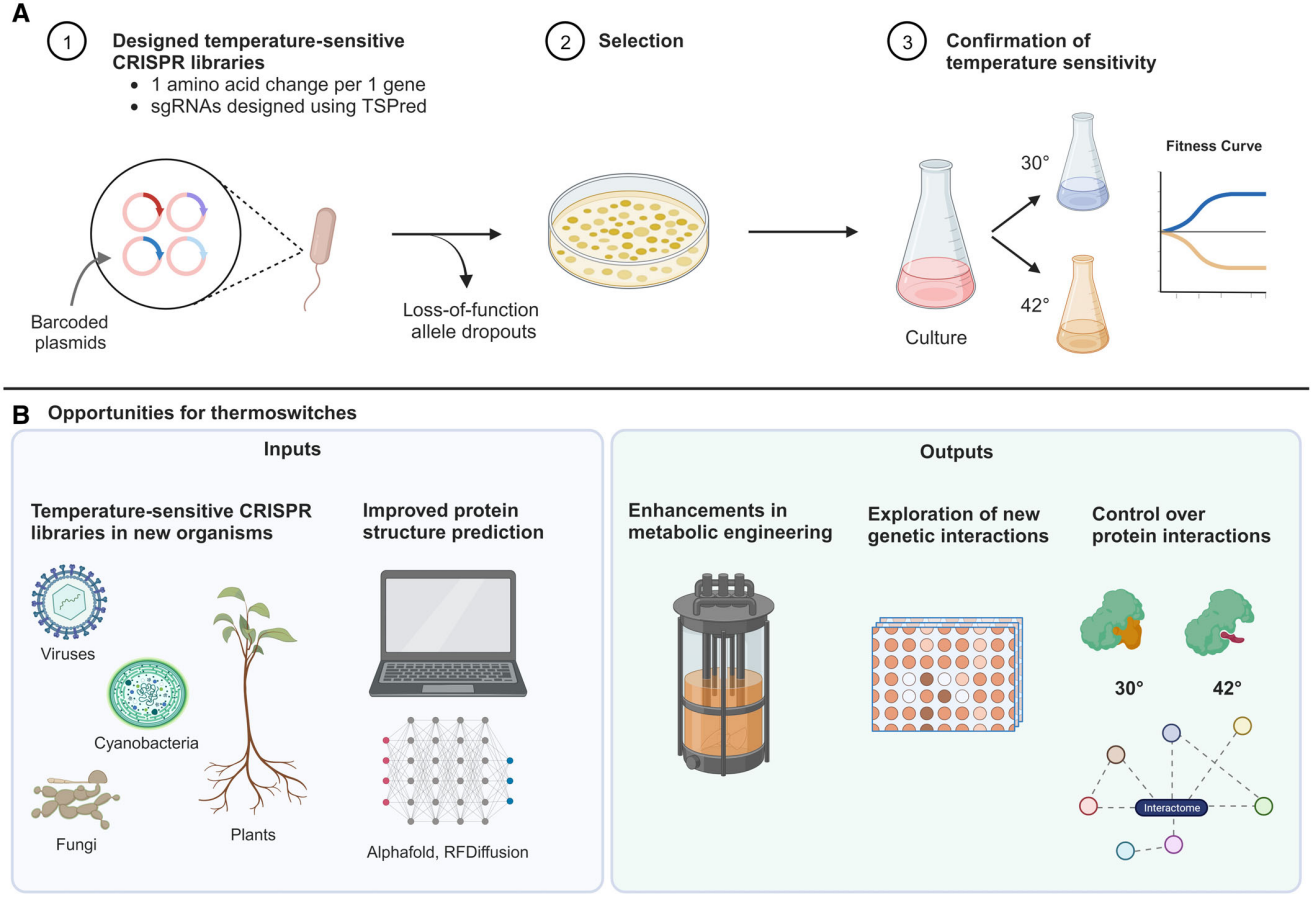
Current and future opportunities in temperature sensitive library design (A) Schematic of thermoswitch library construction by Schramm *et al* (2023). Single‐guide RNAs were designed to introduce single amino acid substitutions in a set of 346 essential *E. coli* genes in minimal glucose medium. The surviving CRISPR library was cultured at permissive (30°C) and nonpermissive (42°C) temperatures. Fitness was measured over time at different temperatures to assess growth and therefore loss of essential protein function. (B) Opportunities for different inputs and outputs of thermoswitch collections. The left panel shows how a similar experimental design can be used to construct thermoswtich libraries in various organisms. This approach will improve with advancements in protein structure prediction. The right panel illustrates potential applications of a growing collection of such libraries that will help enhance metabolic engineering, tailor protein–protein interactions, and expand our understanding of genetic interactions in different organisms.

Efforts to generate TS alleles have been made in many organisms, and they have been most successful and comprehensive in the yeast *Saccharomyces cerevisiae*. In yeast, curation and standardization of decades of published TS alleles along with directed screening methods for new alleles led to a standard collection representing the majority of essential yeast genes (Ben‐Aroya *et al*, 2008; Li *et al*, 2011). In the past decade, this collection has enabled deep mapping of genetic interactions, highlighting the central roles of essential genes in cell biology (Costanzo *et al*, 2010). Suppressor screens and other approaches continue to extend the biological insights derived from the yeast TS alleles (van Leeuwen *et al*, 2020). The study by Schramm *et al* (2023) now provides the tools for performing systematic analyses and generating insights into bacterial systems using TS alleles. Given the role of essential genes as “hubs” in genetic networks, we anticipate significant new biological discoveries to emerge from a deep analysis of the essential bacterial genome.

Building libraries of strains with “thermoswitches” not only holds significant promise for advancing our understanding of biology but also opens avenues for practical applications. TS alleles offer a versatile toolkit for researchers to control and optimize chemical and metabolite production for synthetic biology. Schramm *et al* (2023) demonstrate this concept by decoupling growth from arginine overproduction using a TS allele for DNA Polymerase III gamma/tau DnaX^L289Q^. The authors show that at 42°, the DnaX^L289Q^ mutation causes a growth arrest but the arginine production pathway is still active for the 24 h tested. This offers a new opportunity for chemical production since it inhibits growth while enabling ongoing metabolite production. This approach could in principle also reduce the uptake of nutrients required by growing cultures, making the production of large batches of chemicals more feasible. However, this remains to be tested. During chemical synthesis, maintaining metabolic flux is key and this has been shown successfully in cyanobacteria. For instance, in butanol production, ensuring ample supply of the precursor Acetyl‐CoA is important to generate large outputs. To this end, Liu *et al* (2019) blocked competing pathways by deleting genes to redirect Acetyl‐CoA for the desired butanol production. It is easy to imagine that tunable thermoswitches can be used to control such genes to ensure activation or depletion by regulating the temperature of a bioreactor. Importantly, the workflow described in Schramm *et al* (2023) combined with the TSPred algorithm is not necessarily limited to *E. coli* and could be applied to various industrially useful organisms such as *Pichia pastoris*, cyanobacteria or plants to create synthetic thermoswitch libraries (Fig 1B).

Finally, the dawn of reliable protein structure prediction using alphafold and generative AI (Jumper *et al*, 2021; Watson *et al*, 2023) offers exciting opportunities for engineering‐designed TS alleles. Schramm *et al* (2023) did identify amino acid biases in the selected TS alleles, suggesting that experimental data could be integrated with computational approaches to optimize library design in the future. Improvements in protein design over the coming years will only enhance our understanding of how mutations affect protein stability. Fully understanding the principles that make a good TS allele would enable further tuning of thermoswitches to specifically destabilize activities, interactions, or domains in target proteins (Fig 1B). This could enable temperature‐controlled activation or deactivation of pathways that are dependent on certain protein interactions, thus increasing our control over biological processes. Integration of these computational tools with the experimental mutagenesis and screening pipeline established by Schramm *et al* (2023) could contribute to exciting advances in synthetic biology and metabolic engineering.

## Disclosure and competing interests statement

The authors declare that they have no conflict of interest.
